# Pleiotropic Roles of ChSat4 in Asexual Development, Cell Wall Integrity Maintenance, and Pathogenicity in *Colletotrichum higginsianum*

**DOI:** 10.3389/fmicb.2018.02311

**Published:** 2018-10-24

**Authors:** Ji-Yun Yang, Yu-Lan Fang, Ping Wang, Jian-Ren Ye, Lin Huang

**Affiliations:** Co-Innovation Center for Sustainable Forestry in Southern China, Nanjing Forestry University, Nanjing, China

**Keywords:** *Colletotrichum higginsianum*, serine/threonine kinase, K^+^ accumulation, cell wall integrity, pathogenicity

## Abstract

Potassium has an important role to play in multiple cellular processes. In *Saccharomyces cerevisiae*, the serine/threonine (S/T) kinase Sat4/Hal4 is required for potassium accumulation, and thus, regulates the resistance to sodium salts and helps in the stabilization of other plasma membrane transporters. However, the functions of Sat4 in filamentous phytopathogenic fungi are largely unknown. In this study, ChSat4, the yeast Sat4p homolog in *Colletotrichum higginsianum*, has been identified. Target deletion of *ChSAT4* resulted in defects in mycelial growth and sporulation. Intracellular K^+^ accumulation was significantly decreased in the *ChSAT4* deletion mutant. Additionally, the Δ*Chsat4* mutant showed defects in cell wall integrity, hyperoxide stress response, and pathogenicity. Localization pattern analysis indicated ChSat4 was localized in the cytoplasm. Furthermore, ChSat4 showed high functional conservation with the homolog FgSat4 in *Fusarium graminearum*. Taken together, our data indicated that ChSat4 was important for intracellular K^+^ accumulation and infection morphogenesis in *C. higginsianum*.

## Introduction

Potassium is indispensable for multiple cellular processes, wherein it has important roles in the maintenance of the membrane potential and intracellular pH, and regulation of cellular enzyme activity (Kahm et al., 2012). Potassium is observed to be accumulated against its electrochemical gradient at a higher concentration intracellularly than that in the extracellular environment. In *Saccharomyces* *cerevisiae*, the uptake of potassium across the plasma membrane is driven by the membrane potential, which is generated by proton pumping via H^+^-ATPase (Rodriguez-Navarro, 2000; Buch-Pedersen et al., 2006). Genetic analyses revealed that potassium transport in *S. cerevisiae* is regulated by several proteins, including the high-affinity transporter proteins Trk1 and Trk2; the protein kinases Hal4, Hal5, and Sky1; the protein phosphatase Ppz1, Ppz2, and calcineurin; the G protein Arl1; and a protein of unknown function, Hal1 (Rodriguez-Navarro, 2000; Rotin et al., 2000; Wang et al., 2005; Pérez-Valle et al., 2007). In *S. cerevisiae, SAT4* encodes a serine/threonine kinase, which positively regulates potassium uptake through a Trk1p-dependent manner (Mulet et al., 1999). This protein also contributes to the stabilization of the plasma membrane transporters, the control of carbon and nitrogen metabolism, and the regulation of the transcriptional activator Gln3 (Mulet et al., 1999; Pérez-Valle et al., 2010; Hirasaki et al., 2011). The deletion of *SAT4* resulted in a remarkably lower cellular K^+^ concentration. Consequently, a *sat4* mutant exhibited hypersensitivity to Na^+^, Li^+^, and Ca^2+^ (Mulet et al., 1999). In the filamentous fungus *Fusarium graminearum*, the deletion of *FgSAT4* inhibited hyphal growth and sporulation, altered the normal conidial morphology, increased the sensitivity to NaCl, and decreased the pathogenicity to the host plant (Wang et al., 2011; Zheng et al., 2012). These observations indicated that *SAT4* and its orthologs may share different functions and regulatory mechanisms in *S. cerevisiae* and phytopathogenic fungi.

Anthracnose caused by *Colletotrichum* spp. affects a wide range of commercial crops and plants worldwide (Waller, 1992; Nakamura et al., 2018). *Colletotrichum higginsianum* causes anthracnose and blight on several members of Brassicaceae, and causes significant economic losses (O’Connell et al., 2004). *C. higginsianum* employs a hemibiotrophic infection process, which initially establishes infection through a conidium that germinates and produces a germ tube that further develops into an appressorium, which ruptures the host cuticle, produces specialized primary biotrophic hyphae to invade the host cells, and finally differentiates into necrotrophic hyphae that destroy and kill the host tissues (O’Connell et al., 2004, 2012; Plaumann et al., 2018). Sat4 is involved in the regulation of potassium accumulation and stress resistance in *S. cerevisiae*. Although filamentous fungi have different lifestyles from that of yeast, the functions of SAT4 orthologs in phytopathogenic fungi are largely unknown.

In this study, a yeast S/T kinase Sat4 homolog ChSat4 was identified and characterized in *C. higginsianum*. We show that ChSat4 is not only involved in K^+^ accumulation, but is also important for hyphal growth, sporulation, cell wall integrity, and pathogenicity.

## Materials and Methods

### Fungal Strains and Culture

*Colletotrichum higginsianum* strain IMI349063 was used as the wild type for transformation. All strains were cultured in an incubator at 25°C. Modified Mathur’s medium, oat meal agar (OMA), minimal medium (MM), and potato dextrose agar (PDA) were used to analyze the vegetative growth of the fungal strains (Qi et al., 2012). Liquid complete medium (CM) was used to culture and harvest the fungal mycelia for genomic DNA extraction, and the protoplasts were prepared as described (Sweigard et al., 1992).

### Nucleic Acid Manipulations, Southern Blotting, and Semiquantitative Reverse Transcription-Polymerase Chain Reaction (RT-PCR)

Fungal genomic DNA was extracted as described by Damm et al. (2008). Southern blotting was preformed according to the manufacturer’s instructions of a DIG High Prime DNA Labeling and Detection Starter Kit (Roche Applied Science, Penzberg, Germany). Total RNA was isolated using an RNA extraction kit (Invitrogen, Carlsbad, CA, United States). Semiquantitative RT-PCR was performed as described by Huang et al. (2017).

### Targeted Gene Deletion and Complementation

The *ChSAT4* gene replacement construct was generated using the standard one-step gene replacement strategy (Tang et al., 2015). The upstream and downstream flanking sequences of *ChSAT4* were amplified using primer sets Ch10150_U_F/Ch10150_U_R and Ch10150_D_F/Ch10150_D_R, respectively (Supplementary Table S1). The resulting PCR products were digested with restriction endonucleases and ligated to the hygromycin phosphotransferase (*HPH*) cassette released from the pCX62 vector. After ligation, a 3.4-kb gene replacement fragment was amplified with a primer set Ch10150_U_F/Ch10150_D_R and transformed into the protoplasts of *C. higginsianum* wild type IMI349063 using the method of protoplasts preparation and transformation employed in *F. graminearum* as described by Li et al. (2018).

For complementation assays, a 2.2-kb fragment containing the whole *ChSAT4* gene and its native promoter region was amplified with primers Ch10150_COM_F/R (Supplementary Table S1) and inserted into the vector pYF11 (Zhao et al., 2004). The resulting construct *ChSAT4-GFP* was verified by sequencing and transformed into the Δ*Chsat4* mutant.

To assay the functional conservation of Sat4 between *C. higginsianum* and *F. graminearum*, the construct *ChSAT4-GFP* was transformed into the Δ*Fgsat4* mutant to generate the Δ*Fgsat4/ChSAT4* strain. First, the Δ*Fgsat4* mutant was generated using the split marker approach with the primers FGSG_06939_F1/R2, HYGF/R, and FGSG_06939_F3/R4. After transformation of the wild type PH-1, hygromycin-resistant transformants were screened using PCR with primers sets FGSG_06939_InF/R and FGSG_06939_OuF/HPHCON_R2 (Supplementary Table S1). Second, the construct *ChSAT4-GFP* was transformed into protoplasts of the Δ*Fgsat4* mutant to generate the Δ*Fgsat4/ChSAT4* strains. The transformants resistant to zeomycin were screened using PCR with primer set FGSG_06939_InF/R and GFP signal examination.

### Vegetative Growth, Sporulation, Stress Resistance Assays

Mycelial blocks of IMI349063, the Δ*Chsat4* mutant, and the complemented strain were inoculated onto CM, Mathur’s, PDA, and MM media in the dark at 25°C (Araújo et al., 2014). The diameters of the fungal colonies were measured after incubation for 5 days. The conidia of the wild type IMI349063, the Δ*Chsat4* mutant, and the complemented strain were induced in the CMC liquid as described by Araújo et al. (2014).

For stress resistance assays, mycelial blocks (5 × 5 mm) were inoculated onto Mathur’s agar plates containing H_2_O_2_ (5, 7.5, and 10 mM), NaCl (0.7 M), sodium dodecyl sulfate (SDS) (0.005%), Calcofluor white (CFW) (50 and 100 μg mL^-1^), and Congo red CR (200, 400, and 600 μg mL^-1^), respectively, and cultured at 25°C in the dark for 5 days (Guo et al., 2010). All experiments were carried out three times with three replicates.

### Determination of the K^+^ Concentration in the Mycelia

The wild type strain IMI349063 and the Δ*Chsat4* strain were cultured in liquid CM and Mathur’s medium for 2 days, respectively. Mycelia were harvested and dried using a freeze drier and digested with a solution containing 98% H_2_SO_4_, and 30% H_2_O_2_ was added to restore the colorless, mycelial digestion solution. Next, the digestions were measured using a flame spectrophotometer (Almeida et al., 2015). The experiments were carried out three times with three replicates.

### Plant Infection Assays

For pathogenicity assays, fresh mycelial mats of the wild type IMI349063, the Δ*Chsat4* mutant, and the complemented strain were inoculated onto unwounded leaves and petioles of *Brassica rapa* subsp. *campestris* (Lyu et al., 2016). The penetration abilities of the wild type IMI349063, the Δ*Chsat4* mutant, and the complemented strain were examined using the cellophane membrane technique (He et al., 2017). To quantify fungal biomass *in planta*, DNA was isolated from infected *Brassica chinensis* leaves inoculated by the wild type and the Δ*Chsat4* mutant at 3 days postinoculation (dpi), respectively. The quantitative PCR (qPCR) was used to examine the relative fungal biomass as described by Plaumann et al. (2018). These experiments were performed three times, with three replicates for each treatment.

For the pathogenicity assays of *F. graminearum* wild type PH-1, the Δ*Fgsat4* mutant, and the Δ*Fgsat4/ChSAT4* strain, blocks of PH-1, Δ*Fgsat4*, and Δ*Fgsat4/ChSAT4* were inoculated into the liquid CMC medium to induce sporulation for 3 days. Then, conidia were collected and suspended in ddH_2_O at a concentration of 10^6^ conidia/ml. Ten milliliters of conidial suspension were inoculated into tomato (*Lycopersicon esculentum*) fruits and incubated in a chamber at 25°C (Gao et al., 2016). In order to quantify fungal biomass during infection, DNA was isolated from infected tomato fruit inoculated by the wild type PH-1, the Δ*Fgsat4* mutant, and the transformant Δ*Fgsat4/ChSAT4* at 4 dpi. Relative fungal biomass was monitored using qPCR as described by Plaumann et al. (2018). These experiments were carried out three times with three replicates.

### Light Microscopy and Data Analysis

The chitin deposited in the cell wall was observed using CFW (Sigma, St. Louis, MO, United States) staining as described by Huang et al. (2017). Photographs were taken using a Zeiss M2 microscope (Carl Zeiss, Germany). Statistical analyses were performed with the SPSS 19.0 software program (SPSS Inc., Chicago, IL, United States) using a one-way analysis of variance (ANOVA) (*p* < 0.01).

## Results

### Identification and Deletion of *ChSAT4* in *Colletotrichum higginsianum*

Using the *S. cerevisiae SAT4* sequence as the reference to search the *C. higginsianum* genome database, the CH063_10150 genetic loci encoding the *SAT4* homolog *ChSAT4* was identified. ChSat4 contains 490 amino acids sharing 52% sequence identity with Sat4. Phylogenetic analysis showed that the Sat4 proteins in filamentous fungi have diverged from those of unicellular yeasts, and ChSat4 of *C. higginsianum* and other Sat4 proteins from *Colletotrichum* formed a monophyletic lineage (Supplementary Figure S1A). This result indicates that the Sat4 proteins are conserved in fungi.

In order to investigate the function of ChSat4 in *C. higginsianum*, a gene deletion mutant Δ*Chsat4* was generated by replacing the *ChSAT4* coding region with the hygromycin phosphotransferase resistance (*HPH*) gene. Results showed that the transcript of *ChSAT4* was not detected in the Δ*Chsat4* mutant (Supplementary Figure S1B). Southern blotting analysis further confirmed that the target gene *ChSAT4* was deleted and replaced by *HPH* gene in the Δ*Chsat4* mutant (Supplementary Figure S1C). For mutant complementation, *ChSAT4-GFP* fusion construct was introduced into the Δ*Chsat4* mutant to obtain the complementation strain Δ*Chsat4*/*SAT4* (Supplementary Figure S1B).

### Roles of ChSat4 in Vegetative Growth and Asexual Development

**FIGURE 1:**
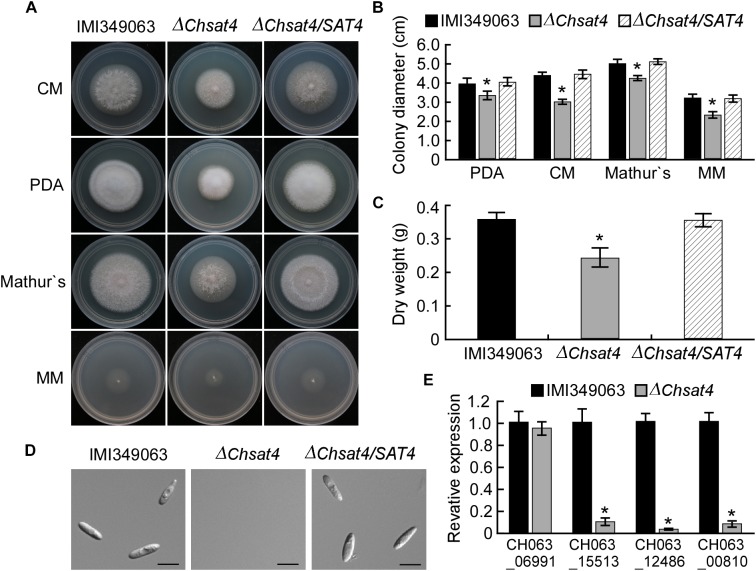
Effects of ChSat4 on vegetative mycelial growth and conidiation. **(A)** Colony morphology of the wild type IMI340963, Δ*Chsat4* mutant, and the complemented strain grown on CM, PDA, Mathur’s, and MM media plates for 5 days. **(B)** Colony diameter of IMI340963, Δ*Chsat4* mutant, and the complemented strain grown on CM, PDA, Mathur’s, and MM media plates for 5 days. **(C)** Dry weight of the mycelia from the wild type IMI340963, Δ*Chsat4* mutant, and the complemented strain. Error bars represent the SD from the means, and asterisks indicate statistically significant differences (*p* < 0.01). **(D)** Sporulation of IMI340963, Δ*Chsat4* mutant, and the complemented strain in CMC medium. Bars = 10 μm. **(E)** The expression levels of conidiation-related genes. The qRT-PCR was performed with the RNA extracted from mycelia of the wild type and Δ*Chsat4* mutant cultured in CM liquid medium for 2 days. Error bars represent the SD, and *t*-test analysis was shown with ^∗^*p* < 0.01 versus the wild type.

To determine the role of ChSat4 in vegetative growth, the wild type IMI349063, the Δ*Chsat4* mutant, and the complemented strain Δ*Chsat4*/*SAT4* were cultured on CM, Mathur’s, PDA, and MM media plates. The Δ*Chsat4* mutant produced smaller colonies compared to the wild type and complemented strains (Figures 1A,B). In addition, the mycelial dry weight of the Δ*Chsat4* mutant was reduced compared to that of the wild type and the complemented strains (Figure 1C).

Asexual spores are important for the disease cycle of *C. higginsianum*. The sporulation abilities of Δ*Chsat4* mutant, the wild type IMI349063, and the complemented strain were assayed. The Δ*Chsat4* mutant did not produce any conidia in the CMC medium, while the wild type and complemented strains produced abundant conidia (Figure 1D). In *Magnaporthe oryzae*, the sporulation is regulated by some genes such as *MoCON7, MoCOM1, MoAPS2* and *MoACR1* (Kim and Lee, 2012; Huang et al., 2017). In order to further analyze the effect of deletion of *ChSAT4* on the expression of conidiation-related genes in *C. higginsianum*, the expression level of homologous of these genes (*CON7*: CH063_06991; *COM1*: CH063_15513; *APS2*: CH063_12486; *ACR1*: CH063_00810) was examined using qRT-PCR. Results showed that the expression levels of these conidiation-related genes was significantly decreased except for the *CON7* in the Δ*Chsat4* mutant compared to the wild type (Figure 1E). These results indicated that the ChSat4 was involved in regulating the vegetative growth and sporulation in *C. higginsianum*.

### Contribution of ChSat4 to K^+^ Accumulation and NaCl Stress Resistance

**FIGURE 2:**
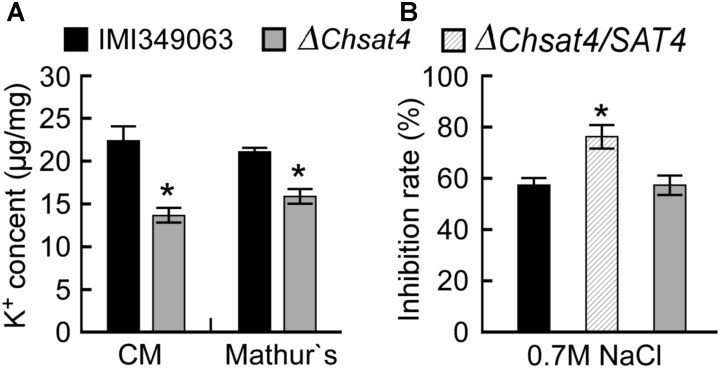
*ChSAT4* deletion mutant decreasing the K^+^ concentration and NaCl stress resistance. **(A)** K^+^ concentration in the mycelia of *C. higginsianum* cultured in CM and Mathur’s media. **(B)** Inhibition rate of mycelial growth of IMI340963, Δ*Chsat4* mutant and the complemented strain grown on CM plates contain 0.7 M NaCl for 5 days. Error bars represent the SD from the means, and asterisks indicate statistically significant differences (*p* < 0.01).

*Saccharomyces cerevisiae* Sat4 was involved in the cellular K^+^ accumulation (Kahm et al., 2012). To investigate whether ChSat4 exhibits a similar function, the concentration of K^+^ in the mycelia of the wild type and the Δ*Chsat4* mutant were examined. In both media of CM and Mathur’s, the K^+^ concentration of the Δ*Chsat4* mutant was significantly decreased compared to that of the wild type (Figure 2A). The K^+^ accumulation may lead to a relatively low osmotic potential, which could cause sensitivity to extracellular osmotic stress. To confirm this hypothesis, the Δ*Chsat4* mutant was exposed to NaCl and the growth inhibition rates were calculated. The results showed that the growth of the Δ*Chsat4* mutant was significantly inhibited compared to that of the wild type and complemented strains (Figure 2B). These results indicated that ChSat4 was involved in the cellular K^+^ accumulation and osmotic stress resistance.

### Effect of Deletion of *ChSAT4* on Cell Wall Integrity

**FIGURE 3:**
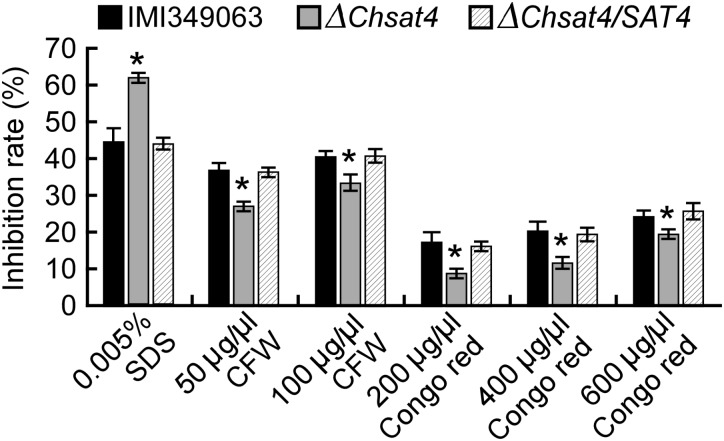
The *CHSAT4* deletion mutant altering the resistance to various cell wall inhibitors. Inhibition rates of hyphal growth of IMI340963, the Δ*Chsat4* mutant, and the complemented strain grown on CM plates containing SDS, CFW, Congo red for 5 days.

To evaluate the effect of the deletion of *ChSAT4* on the cell wall integrity in *C. higginsianum*, the wild type IMI349063, the Δ*Chsat4* mutant, and the complemented strain were inoculated onto Mathur’s plates containing the cell wall inhibitor SDS, CFW, and CR, respectively. When exposed to SDS, the Δ*Chsat4* mutant was more sensitive than the wild type and the complemented strain (Figure 3). However, the Δ*Chsat4* mutant was more tolerant to CFW and CR than the wild type and the complemented strain (Figure 3). These data indicated that the deletion of *ChSAT4* altered the normal resistance to cell wall inhibitors.

**FIGURE 4:**
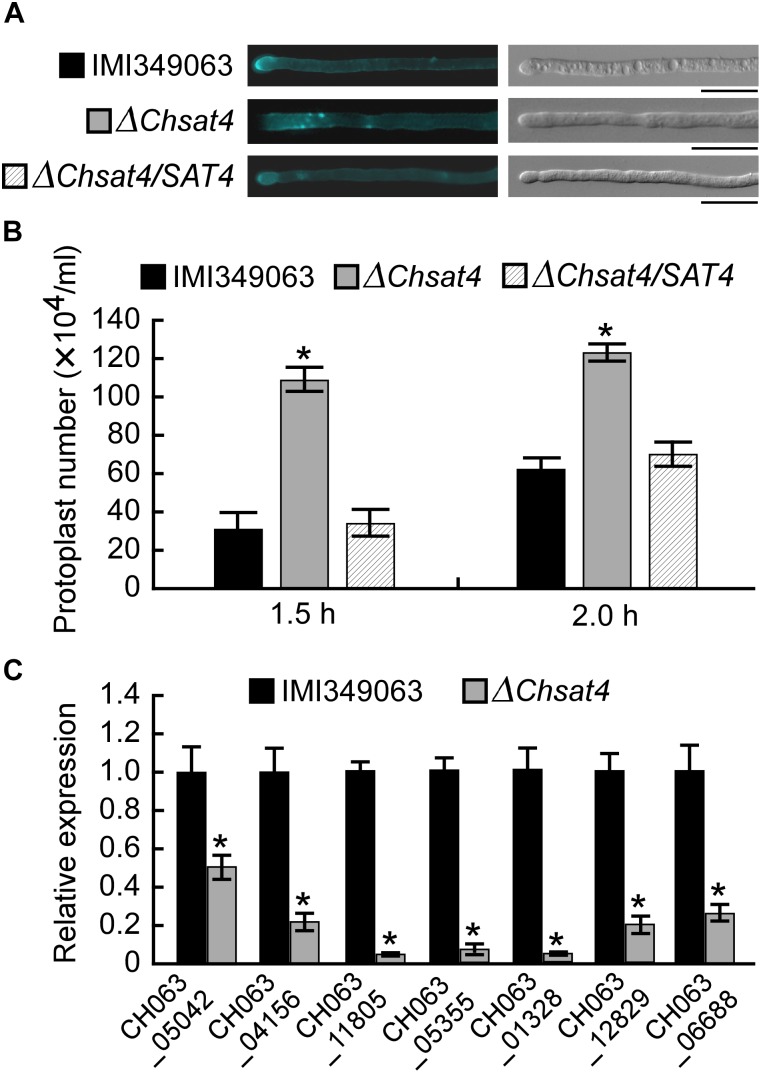
Deletion of *ChSAT4* altering the cell wall integrity. **(A)** Disruption of *ChSAT4* altered the chitin distribution on the cell wall. **(B)** Protoplasts of the wild type and Δ*Chsat4* mutant after being treated with a solution of cell wall lytic enzyme for 1.5 and 2 h. Error bars represent the SD from the means, and asterisks indicate statistically significant differences (*p* < 0.01). **(C)** The expression of seven chitin synthases in the wild type and Δ*Chsat4* mutant. Error bars represent the SD, and *t*-test analysis was shown with ^∗^*p* < 0.01 versus the wild type.

Chitin is a major component of the cell wall of fungi, and the normal synthesis and distribution of chitin are important for polar hyphal tip growth and hyphal morphology (Huang et al., 2017). Chitin distribution was assayed using CFW staining, and results showed that chitin primarily accumulated in hyphal tips in both of the wild type and complemented strain. However, the chitin distribution was not restricted to the growing apices in the Δ*Chsat4* mutant (Figure 4A). To further analyze the effect of deletion of *ChSAT4* on the cell wall integrity, the protoplast release assay was performed, and the results showed that the Δ*Chsat4* mutant released protoplasts more quickly than both of the wild type and complemented strain (Figure 4B). Additionally, the expression level of seven chitin synthases genes (*CHS1*: CH063_05042; *CHS2*: CH063_04156; *CHS3*: CH063_11805; *CHS4*: CH063_05355; *CHS5*: CH063_01328; *CHS6*: CH063_12829; *CHS7*: CH063_06688) was tested using qRT-PCR. The result showed that the expression levels of these seven genes were significantly lower in Δ*Chsat4* mutant than those of the wild type (Figure 4C). These results indicated that the Δ*Chsat4* mutant was defective in cell wall integrity.

### Function of ChSat4 in Responses to Hyperoxide Stress

**FIGURE 5:**
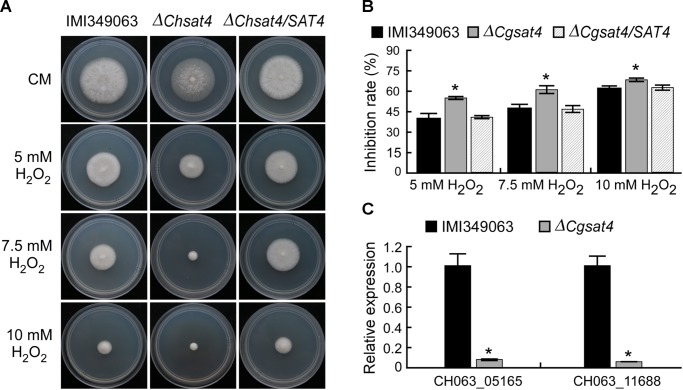
Sensitivity of the *ChSAT4* deletion mutant to H_2_O_2_. **(A)** Colony diameter of IMI340963, Δ*Chsat4* mutant, and the complemented strain grown on CM plates containing different concentrations of H_2_O_2_ for 5 days. **(B)** Inhibition rates of mycelial growth of IMI340963, Δ*Chsat4* mutant, and the complemented strain exposed to H_2_O_2_. Error bars represent SD, and asterisks indicate statistically significant differences (*p* < 0.01). **(C)** The expression levels of catalase and peroxidase in the wild type and the Δ*Chsat4* mutant. Error bars represent the SD, and *t*-test analysis was shown with ^∗^*p* < 0.01 versus the wild type.

To evaluate the effect of the *ChSAT4* deletion on the resistance to oxidative stress, the wild type, the Δ*Chsat4* mutant, and the complemented strain were inoculated onto Mathur’s plates where they were subjected to H_2_O_2_. In the presence of H_2_O_2_, the Δ*Chsat4* mutant showed higher growth inhibition rates than those of the wild type and complemented strain (Figures 5A,B). The qRT-PCR analysis showed that the expression levels of catalase (CAT: CH063_11688) and peroxidase (POX: CH063_05165) were significantly decreased in the Δ*Chsat4* mutant (Figure 5C). These results indicated that the deletion of *ChSAT4* may result in the reduced expression of the genes involved in reactive oxygen species scavenging, and lead to a greater sensitivity to H_2_O_2_.

### Pathogenicity of the *ChSAT4* Deletion Mutant

**FIGURE 6:**
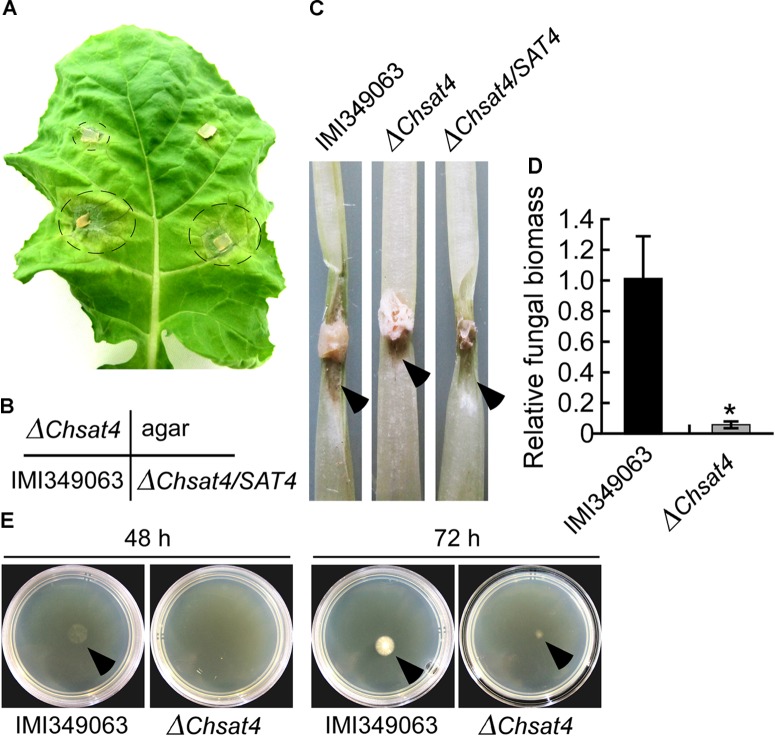
Defects of the Δ*Chsat4* mutant in pathogenicity to the host plant. **(A)** Pathogenicity assay of the wild type IMI340963, Δ*Chsat4* mutant, and the complemented strain on detached leaves of *Brassica rapa* subsp. *campestris*. Circles indicate the diseased areas. **(B)** The plug position of the wild type IMI340963, Δ*Chsat4* mutant, and the complemented strain, and a blank CM plug in **(A)** was indicated. **(C)** Pathogenicity assay when inoculation of wild type IMI340963, Δ*Chsat4* mutant, and the complemented strain on detached petioles of *Brassica rapa* subsp. *campestris*. Lesions are indicated by arrows. **(D)** Relative fungal biomass monitored from infected tissues of *B. chinensis* at 3 dpi. The qPCR was performed to amplify the *C. higginsianum actin* DNA gene relative to the *B. chinensis actin* gene. Error bars represent the SD, and *t*-test analysis was shown with ^∗^*p* < 0.01 versus the wild type. **(E)** Penetration of cellophane sheets. Colonies of the wild type IMI349063 and the Δ*Chsat4* mutant grown for 48 and 72 h on a CM medium plate covered by a cellophane sheet (L), and the colony in the same plate with cellophane removal and incubated for one additional day (R), respectively. Colonies are indicated by arrows.

Since the Δ*Chsat4* mutant was defective in sporulation, mycelial blocks of the wild type, the Δ*Chsat4* mutant, and the complemented strain were inoculated on detached leaves and petioles of *B. rapa* subsp. *campestris*, respectively, to evaluate their pathogenicity. At 5 dpi, typical and enlarged lesions were observed on the leaves of *B. rapa* subsp. *campestris* inoculated with the wild type and complemented strains. In contrast, there were very small and limited lesions on the leaves inoculated with the Δ*Chsat4* mutant. There were no lesions observed on the leaves inoculated solely with agar (Figures 6A,B). When the petioles were inoculated using the mycelial mats of the wild type, the Δ*Chsat4* mutant, and the complemented strain, similar results were observed (Figure 6C). In order to evaluate differences in fungal biomass between the Δ*Chsat4* mutant and the wild type during infection, the *C. higginsianum actin* DNA fragment was amplified by qPCR using the *B. rapa actin* gene for normalization. The result showed that a highly significant reduction in the amount of fungal actin DNA was observed at 3 dpi for the Δ*Chsat4* mutant compared with the wild type (Figure 6D).

The *in vitro* cellophane penetration ability has been reported to correlate significantly with *in vivo* pathogenicity in *Fusarium oxysporum* and other filamentous fungi (Rosales and Pietro, 2008; Rispail and Di Pietro, 2009; Gu et al., 2015). To further investigate the role of ChSat4 in mycelial penetration ability, the wild type and Δ*Chsat4* mutant were inoculated on a cellophane membrane placed on a CM plate, respectively. The Δ*Chsat4* mutant penetrated the cellophane and formed a colony on the medium plate at 72 h postinoculation (hpi). In contrast, the wild type was able to cross cellophane membranes at 48 hpi (Figure 6E). These results indicated that ChSat4 was required for the mycelial penetration and full virulence in *C. higginsianum*.

### Subcellular Localization of ChSat4-GFP Fusion Protein

**FIGURE 7:**
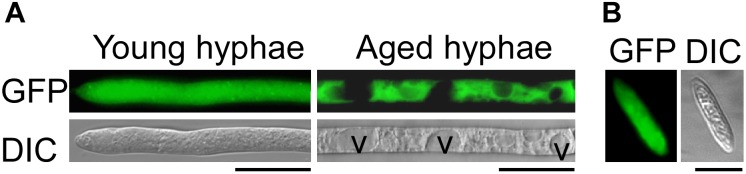
Cellular localization of ChSat4-GFP. The subcellular localization of ChSat4 detected using a *ChSAT4-GFP* fusion protein driven by its native promoter. **(A)** Cellular localization of ChSat4-GFP in vegetative hyphae. V indicates the vesicles. **(B)** Cellular localization of ChSat4-GFP in conidia. Bars = 10 μm.

The *ChSAT4-GFP* fusion construct was introduced into the Δ*Chsat4* mutant, and the complementation strain Δ*Chsat4*/*SAT4* was obtained. Our data indicated that the complemented strain Δ*Chsat4/SAT4* completely restored the defects in the Δ*Chsat4* mutant as shown earlier. To further investigate the localization pattern of the ChSat4 in *C. higginsianum*, GFP signal of the fusion protein ChSat4-GFP expressed in the Δ*Chsat4/SAT4* strain was observed under an epifluorescence microscope. Results showed that a strong green fluorescence signal was observed in the hyphal cytoplasm in the Δ*Chsat4/SAT4* strain (Figure 7A). We also observed the same GFP localization pattern in the conidia (Figure 7B). These results suggest that ChSat4 was localized in the cytoplasm in *C. higginsianum*.

### Cross-Species Function of ChSat4 With FgSat4 in *F. graminearum*

**FIGURE 8:**
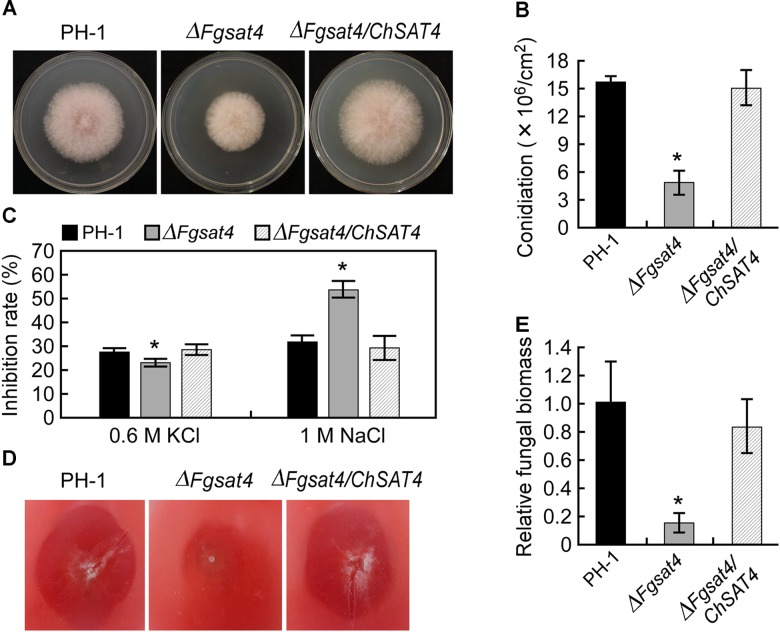
Restoration of some defects of the Δ*Fgsat4* mutant in *Fusarium graminearum* by ChSAT4. **(A)** Mycelial growth of the wild type PH-1, Δ*Fgsat4* mutant, and Δ*Fgsat4/ChSAT4* strain on CM medium plates. **(B)** The ability to produce conidia of wild type PH-1, Δ*Fgsat4* mutant, and Δ*Fgsat4/ChSAT4* strain. **(C)** The mycelial growth inhibition rate of the wild type PH-1, Δ*Fgsat4* mutant, and Δ*Fgsat4/ChSAT4* strain when exposed to NaCl and KCl. **(D)** Pathogenicity of wild type PH-1, Δ*Fgsat4* mutant, and Δ*Fgsat4/ChSAT4* strain on tomato fruit at 5 dpi. **(E)** Relative fungal biomass during infection monitored by qPCR. Total DNA was extracted from leaf tissues inoculated either with the wild type PH-1, Δ*Chsat4* mutant, and Δ*Fgsat4/ChSAT4* strain at 4 dpi. Error bars represent SD, and asterisks indicate statistically significant differences (*p* < 0.01).

Like *C. higginsianum, F. graminearum* belongs to hemibiotrophic phytopathogen, employing a biotrophic phase during its initial stage of infection before switching to necrotrophy and inducing cell death (Jansen et al., 2005; Huang et al., 2016). To assess the functional conservation of the Sat4 protein in the filamentous fungi *C. higginsianum* and *F. graminearum*, the *ChSAT4-GFP* fusion construct was introduced into the Δ*Fgsat4* mutant and produced the transformant Δ*Fgsat4/ChSAT4*. The mycelial growth of the Δ*Fgsat4* mutant was significantly reduced on the CM plates compared to that of the wild type PH-1; however, the transformant of Δ*Fgsat4/ChSAT4* showed similar colony diameter to the wild type (Figure 8A). The ability to produce conidia of the Δ*FgSat4* mutant was significantly decreased, but there was no significant difference between the Δ*Fgsat4/ChSAT4* strain and the wild type (Figure 8B). When exposed to the stressors KCl and NaCl, Δ*Fgsat4/ChSAT4* showed an inhibition rate similar to that of the wild type PH-1 and restored the defects in the resistance to KCl and NaCl shown in the Δ*Fgsat4* mutant (Figure 8C). The pathogenicity assay on tomato fruits also showed that the virulence of the Δ*FgSat4* mutant significantly decreased, but the Δ*Fgsat4/ChSAT4* strain showed strong virulence similar to that of the wild type (Figure 8D). Fungal biomass was monitored during infection, and results showed that the amount of fungal *actin* gene DNA was significantly reduced in the Δ*Fgsat4* mutant than that in the wild type and the Δ*Fgsat4/ChSAT4* strain (Figure 8E). These results suggested that ChSat4 shared some cross-species function with FgSat4 in *F. graminearum*.

## Discussion

In eukaryotic organisms, reversible protein phosphorylation by protein kinases is involved in the regulation of various growth and developmental processes and responses to environmental stimuli (Serrano, 1996). Protein kinase genes, ChCBK1 (Schmidpeter et al., 2017) and ChMK1 (Wei et al., 2016), were important for plant infection in *C. higginsianum*. In this study, the deletion of serine/threonine kinase *ChSAT4* resulted in various defects in *C. higginsianum*, including the reduction of vegetative growth, stress response, K^+^ accumulation, cell wall integrity, and pathogenicity in *C. higginsianum*.

The cell wall of fungi plays a key role in the exchange of material between the cell and the external environment. In plant pathogenic fungi, cell wall integrity is required for polarized hyphal growth, invasive structure development, and the infection of the plant (Samalova et al., 2017). Chitin is a major component of the filamentous fungal cell wall, and the normal synthesis and distribution of chitin are important to maintain polar hyphal tip growth and morphology. The deletion of *ChSAT4* altered the resistance to the cell wall stressors SDS, CFW, and CR. The Δ*Chsat4* mutant was sensitive to SDS and showed stronger resistance to CFW and CR than the wild type and the complemented strain. The CFW staining also showed that the deletion of *ChSAT4* altered the normal distribution of chitin in the mycelium. In addition, when the mycelia were treated with the cell wall lytic enzyme, the Δ*Chsat4* mutant released protoplasts more quickly than the wild type. The expression levels of chitin synthases were significantly inhibited in the Δ*ChSat4* mutant. These data indicated that ChSat4 was involved in the cell wall integrity in *C. higginsianum* by regulating the chitin synthases expression.

Monovalent cations such as protons, potassium, and sodium play multiple key roles in eukaryotic cells (Robinson et al., 1992). The regulation of cellular ion homeostasis is a basic property of living cells (Dichtl et al., 1997). The Na^+^ and K^+^ are important intracellular cations, and the concentration of these cations affects fundamental physiological progresses (Benito et al., 2011). A high concentration of cations usually leads to toxicity. However, the threshold for the toxicity of lithium and sodium is much lower than that for potassium since large amounts of sodium and lithium accumulation in the cytosol tends to destroy essential and sensitive enzymes (Pérez-Valle et al., 2007; Corratgé-Faillie et al., 2010; Hirasaki et al., 2011). In *S. cerevisiae*, the high-affinity potassium uptake system is encoded by the transporters Trk1 and Trk2 (Garciadeblas et al., 2007; Casado et al., 2010). The Trk1–Trk2 system is activated by two protein kinases encoded by two paralogs, Sat4/Hal4 and Hal5, to increase the influx of potassium and decrease the membrane potential toxicity (Pérez-Valle et al., 2010). The Δ*Scsat4* mutant was extremely sensitive to a variety of toxic cations such as lithium and sodium (Munson et al., 2004). In *F. graminearum*, FgSat4 was not directly involved in osmoregulation, but it may be specifically involved in the regulation of K^+^/Na^+^ transporter (Wang et al., 2011). In this study, the deletion of *ChSAT4* significantly decreased the concentration of intracellular K^+^ in *C. higginsianum*. In addition, the Δ*Chsat4* mutant altered the resistance to KCl and NaCl in the extracellular environment. These results indicated that ChSat4 was required for the regulation of the intracellular and extracellular balance of K^+^, and the deletion of *ChSAT4* could change the normal physiological process. Whether ChSat4 regulates the accumulation K^+^ to maintain the proper intracellular concentration, and avoid cation toxicity by regulating the activity of K^+^ transporter needs to be explored in further research.

Multifaceted signaling pathways are involved in plant-pathogen interaction (Imam et al., 2016b; Kumar et al., 2016). In plants, the rapid accumulation of ROS is considered to be the first response against invading pathogens (Shetty et al., 2007; Imam et al., 2016a). Hydrogen peroxide (H_2_O_2_), an important ROS, has been reported to inhibit biotrophic pathogens, but to benefit necrotrophic pathogens (Mellersh et al., 2002; Able, 2003). In the hemibiotrophic fungus, *Septoria tritici*, hyphal growth was inhibited by H_2_O_2_ during the biotrophic phase, but a large H_2_O_2_ accumulation occurs in the host during reproduction (Shetty et al., 2007). *C. higginsianum* develops large bulbous biotrophic hyphae in the first infected cell, and the necrotrophic secondary filamentous hyphae develop in the neighboring cells (Korn et al., 2015). During these plant infection processes, *C. higginsianum* needs to counteract the ROS stress and regulate gene expression in response to oxidative stress. In this study, the Δ*Chsat4* mutant showed a higher mycelial growth inhibition rate than the wild type and complemented strain when exposed to H_2_O_2_. Since H_2_O_2_ is involved in the host defense in the early interaction between *C. higginsianum* and its host (Huser et al., 2009), the defect of resistance to H_2_O_2_ may lead to a reduced ability to scavenge host-derived ROS and an attenuation of virulence.

As a homolog of ChSat4, FgSat4 (Fg06939) was identified in the phytopathogenic fungus *F. graminearum* (Wang et al., 2011). The deletion of *FgSAT4* resulted in a defect in resistance to NaCl stress (Wang et al., 2011). *C. higginsianum ChSAT4* shares a 75% sequence identity with *FgSAT4* in *F. graminearum* (Supplementary Figure S2). Sequence analysis also showed that both of *FgSAT4* and *ChSAT4* have a conserved STKc_HAL4_like domain (Supplementary Figure S2), which was determined to regulate potassium ion uptake and cellular resistance to other ions such as sodium in budding and fission yeast (Marchler-Bauer et al., 2017). Here, our data showed ChSat4 is required for potassium accumulation and resistance to ion stress in *C. higginsianum* (Figure 2). Heterologous expression of ChSAT4 in the Δ*Fgsat4* mutant restored its defects in stress resistance to KCl and NaCl (Figure 7). These results may indicate that Sat4 not only has a conserved function in potassium uptake and cation stress resistance in yeast, but also shares the similar roles in phytopathogenic fungi. Furthermore, the deletion of *FgSAT4* significantly reduced the ability to produce conidia, and resulted in the defect in pathogenicity to the host plant in *F. graminearum* (Wang et al., 2011). However, in *C. higginsianum*, conidiation was completely abolished, and the virulence to the host was significantly reduced in the Δ*Chsat4* mutant (Figure 6). Interestingly, expression of ChSAT4 in the Δ*Fgsat4* mutant restored the ability to produce conidia and pathogenicity in the Δ*Fgsat4* mutant (Figure 8). These results may indicate that Sat4 shares a conserved regulation to pathogenicity in *C. higginsianum* and *F. graminearum*. Whether the kinase Sat4 is involved in regulating pathogenicity in other plant pathogenic fungi and its expression regulation model needs to be emphasized in further studies.

## Conclusion

Serine/threonine kinase ChSat4 is required for vegetative growth, asexual development, stress response, K^+^ accumulation, cell wall integrity, and full pathogenicity in *C. higginsianum*.

## Author Contributions

LH and J-RY conceived and designed the experiments. J-YY, Y-LF, and PW performed the experiments. LH, J-YY, and Y-LF analyzed the experiment data. LH and J-RY contributed to reagents, materials, and analysis tools. LH and J-YY wrote the paper. All authors have read and approved of the final manuscript.

## Conflict of Interest Statement

The authors declare that the research was conducted in the absence of any commercial or financial relationships that could be construed as a potential conflict of interest.
