# Supplementary material for: Pleiotropic Roles of ChSat4 in Asexual Development, Cell Wall Integrity Maintenance, and Pathogenicity in Colletotrichum higginsianum
Source: Front Microbiol. 2018 Oct 24;9:2311. doi: 10.3389/fmicb.2018.02311 (PMC6208185; doi:10.3389/fmicb.2018.02311)
Supplement: Supplementary file 1 [file Data_Sheet_1.pdf]

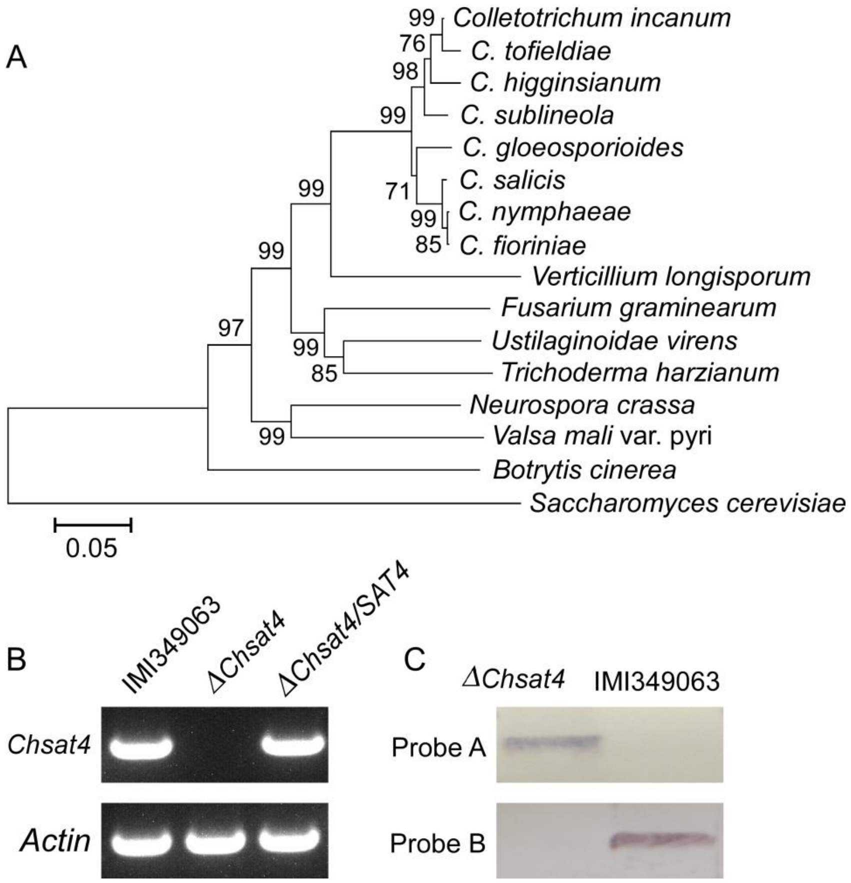

FIGURE S1. Phylogenetic analysis of ChSAT4 and its homologs from different organisms and targeted gene deletion and complementation. **(A)** Phylogenetic tree of Sat4 proteins was constructed based on alignment of the full sequences of Sat4 from different fungi species: *Colletotrichum incanum* (KZL85620), *C. tofieldiae* (KZL70679), *C. sublineola* (KDN68469), *C. gloeosporioides* (ELA23866), C. *salicis* (KXH66554), *C. nymphaeae* (KXH61442), *C. fioriniae* (EXF77336), *Verticillium longisporum* (CRK30296), *Fusarium graminearum* (XP_011326608), *Ustilaginoidae virens* (KDB17453), *Trichoderma harzianum* (KKO97119), *Neurospora crassa* (XP_962991), *Valsa mali* var. pyri (KUI59819), *Botrytis cinerea* (CCD44899), *Saccharomyces cerevisiae* (NP_009934). Phylogenetic tree of ChSat4 homologues from several other species was obtained using neighbor-joining method in Mega7.0 Beta program. An interior-branch test using 1,000 bootstrap replicates was used to evaluate the relative stability of the branches. **(B)** Semi-quantitative RT-PCR analysis of the expression of *ChSAT4* in the deletion and reintroduction of the strains. Data comprise three independent experiments with triple replications that yielded similar results. **(C)** Southern blot analysis of the Δ*Chsat4* mutant. Probe A, the hybridization probe generated from *HPH* gene. Probe B, the hybridization probe generated from *ChSAT4*.

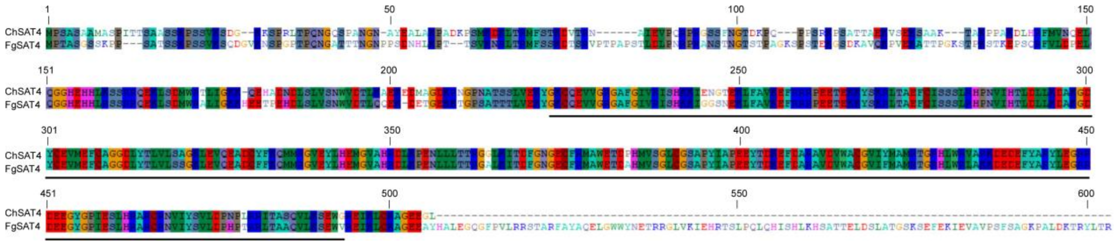

FIGURE S2. Alignment analysis of ChSAT4 and FgSAT4. Conserved amino acids were shaded, and conserved STKc_HAL4_like domains were underlined. Sequenes were retrieved form NCBI: ChSAT4 (XP_018162139, *Colletotrichum higginsianum*), and FgSAT4 (XP_011326608, *Fusarium graminearum*).
