# Supplementary material for: Pleiotropic Roles of ChSat4 in Asexual Development, Cell Wall Integrity Maintenance, and Pathogenicity in Colletotrichum higginsianum
Source: Front Microbiol. 2018 Oct 24;9:2311. doi: 10.3389/fmicb.2018.02311 (PMC6208185; doi:10.3389/fmicb.2018.02311)
Supplement: Supplementary file 2 [file Data_Sheet_2.pdf]

TABLE S1 Primers used in this study.

Primer name	Sequences (5'-3')	Remark
Ch10150_U_F	CCGCTCGAGCCCTTCCTTCTCTTTCTACC	*ChSAT4* deletion vector construction
Ch10150_U_R	CCCAAGCTTCTTGTCGGTGCCATTAAAG	*ChSAT4* deletion vector construction
Ch10150_D_F	GGGTCTAGATCATCTACTCTGTCCTGGAT	*ChSAT4* deletion vector construction
Ch10150_D_R	TCCCCGCGGAGTTGTGTCTGGCGAATG	*ChSAT4* deletion vector construction
Ch10150_IN_F	TCATCGGCAAGAAGCAGGAG	*ChSAT4* mutant PCR screening assay and amplification of *ChSAT4* probe for southern blotting
Ch10150_IN_R	CGCAGAGACCAGACACCATAT	Δ*Chsat4* mutant PCR screening assay and amplification of *ChSAT4* probe for southern blotting
Ch10150_OU_F	ACAGACCGCTGAACGCTAC	Δ*Chsat4* mutant PCR screening assay
FGSG_06939_1F	TACTAACCTACCTGCCTCA	Knockout of *FgSAT4*
FGSG_06939_2R	TTGACCTCCACTAGCTCCAGCCAAGCCGCCGCTAATAA GAACAAGAT	Knockout of *FgSAT4*
FGSG_06939_3F	GAATAGAGTAGATGCCGACCGCGGGTTAGCGATGAGT TGGATACAG	Knockout of *FgSAT4*
FGSG_06939_4R	CCGTGCCGAATATGAAGA	Knockout of *FgSAT4*
HYG/F	TTGACCTCCACTAGCTCCAGCCAAGCC	Knockout of *FgSAT4*
HYG/R	GAATAGAGTAGATGCCGACCGCGGGTT	Knockout of *FgSAT4*
FGSG_06939_InF	TGGCAAGAAGCACGAGGAA	Δ*Fgsat4* mutant PCR screening assay
FGSG_06939_InR	ATACTCAACACCACGCATCATC	Δ*Fgsat4* mutant PCR screening assay
FGSG_06939_OuF	GGCATATAGAGCGGCATGATTA	Δ*Fgsat4* mutant PCR screening assay
HPHCON_R2	TCAGGTCGGAGAGCTGTC	Δ*Fgsat4* mutant PCR screening assay
Ch10150_COM_F	TTTCGTAGGAACCCAATCTTCAAAGTTATTGTCTTCCC GTTG	complemented vector construction of *ChSAT4*
Ch10150_COM_R	CACCACCCCGGTGAACAGCTCCTCGCCCTTGCTCACAA GACCCTCTTCACCTGCCT	complemented vector construction of *ChSAT4*
GFP_R	GACACGCTGAACTTGTGGCCGTT	complemented vector construction of *ChSAT4*
FL111	GGAGGTCAACACATCAATG	amplification of *HPH* probe for southern blotting
FL112	CTCTATTCCTTTGCCCTG	amplification of *HPH* probe for southern blotting
Ch01222F	CTATCCTTACTACCCACACG	semi-quantitative RT-PCR and qRT-PCR primer of *ChACTIN*
Ch01222R	AGGTTCTCGTAGTTAGGGC	semi-quantitative RT-PCR and qRT-PCR primer of *ChACTIN*
Ch10150_Q_F	ACGACCTGTCCCTTGTTTC	semi-quantitative RT-PCR primer of *ChSAT4*
Ch10150_Q_R	GCAGATTCTCGGGTTTC	semi-quantitative RT-PCR primer of *ChSAT4*

Ch11688_Q_F	CTGGTACCTACCGCGTTCAT	quantitative RT-PCR primer of *CAT*
Ch11688_Q_R	GAAACCAGCAGTCTGGAAGC	quantitative RT-PCR primer of *CAT*
CH063_05165_Q_F	GTTGCCAGGACGGAGTATGT	quantitative RT-PCR primer of *POX*
CH063_05165_Q_F	GGTCCAGCTCGAAGAAGATG	quantitative RT-PCR primer of *POX*
CH063_05042_Q_F	ACGGTCGTGCCAAGATTAAC	quantitative RT-PCR primer of *CHS1*
CH063_05042_Q_ R	CTTAGGGACCAAGGTGACCA	quantitative RT-PCR primer of *CHS1*
CH063_04156_Q_F	CGAGCTCTCCTTTGGACAAC	quantitative RT-PCR primer of *CHS2*
CH063_04156_Q_ R	CATCGAAAGTCGGGGATAGA	quantitative RT-PCR primer of *CHS2*
CH063_11805_Q_F	AGGCATCGAGACATGGATTC	quantitative RT-PCR primer of *CHS3*
CH063_11805_Q_ R	GAGATTGTTAACGGCCAGGA	quantitative RT-PCR primer of *CHS3*
CH063_05355_Q_F	GAGAAGAGCCTGGAGGAGGT	quantitative RT-PCR primer of *CHS4*
CH063_05355_Q_ R	GTTGATGGCCAGCTTCTCTC	quantitative RT-PCR primer of *CHS4*
CH063_01328_Q_F	TTCCAAGTGCTGTCGTCAAG	quantitative RT-PCR primer of *CHS5*
CH063_01328_Q_ R	TCGAGAACCTGCTTGATCCT	quantitative RT-PCR primer of *CHS5*
CH063_12829_Q_F	ATCCTCCTCACCTCCTCCAT	quantitative RT-PCR primer of *CHS6*
CH063_12829_Q_ R	CCACGAGTTCTCGTCGTACA	quantitative RT-PCR primer of *CHS6*
CH063_06688_Q_F	CTCCAAGGGTTCCAAGTACG	quantitative RT-PCR primer of *CHS7*
CH063_06688_Q_ R	GCTGTTGGCGTAGTTCACAA	quantitative RT-PCR primer of *CHS7*
CH063_06991_Q_F	ACGCCAGAAGAGTTCAAGGA	quantitative RT-PCR primer of *CON7*
CH063_06991_Q_R	GTACTGCGAAGGCTGGTAGC	quantitative RT-PCR primer of *CON7*
CH063_15513_Q_F	TCCGACAGTTCGACAAGATG	quantitative RT-PCR primer of *COM1*
CH063_15513_Q_R	CGGGTATCCCTGACCTTGTA	quantitative RT-PCR primer of *COM1*
CH063_00810_Q_F	ACATCACGAGGAAGGTGTCC	quantitative RT-PCR primer of *ACR1*
CH063_00810_Q_R	GCACGAGAGTAGTGCTGCTG	quantitative RT-PCR primer of *ACR1*
CH063_12486_Q_F	GATCAACGCAACACACATCC	quantitative RT-PCR primer of *APS2*
CH063_12486_Q_R	GCCTGGGGTGTATTCAAAGA	quantitative RT-PCR primer of *APS2*
BcActin_Q_F	GTCCT GTTCC AGCCT TCGTT C	qPCR primer of *actin* in *B. chinensis*
BcActin_Q_R	CAAGT CCTTC CTGAT ATCCA CGTC	qPCR primer of *actin* in *B. chinensis*
LeActin_Q_F	CGATGTGTGATCTCCTATGGTC	qPCR primer of *actin* in *L. esculentum*
LeActin_Q_R	AGCTGATGGGCTCTAGAAATC	qPCR primer of *actin* in *L. esculentum*
FgActin_Q_F	ATGGTGTCACTCACGTTGTCC	qPCR primer of *actin* in *F. graminearum*
FgActin_Q_R	CAGTGGTGGAGAAGGTGTAACC	qPCR primer of *actin* in *F. graminearum*
